# Application of Plant Viruses in Biotechnology, Medicine, and Human Health

**DOI:** 10.3390/v13091697

**Published:** 2021-08-26

**Authors:** Srividhya Venkataraman, Kathleen Hefferon

**Affiliations:** Department of Cell and Systems Biology, University of Toronto, Toronto, ON M5S 3B2, Canada; byokem@hotmail.com

**Keywords:** expression vectors, aspect ratio, VLPs, VNPs, TMV, PVX, CPMV, geminivirus, cancer, theranostics, CRISPR-cas9

## Abstract

Plant-based nanotechnology programs using virus-like particles (VLPs) and virus nanoparticles (VNPs) are emerging platforms that are increasingly used for a variety of applications in biotechnology and medicine. Tobacco mosaic virus (TMV) and potato virus X (PVX), by virtue of having high aspect ratios, make ideal platforms for drug delivery. TMV and PVX both possess rod-shaped structures and single-stranded RNA genomes encapsidated by their respective capsid proteins and have shown great promise as drug delivery systems. Cowpea mosaic virus (CPMV) has an icosahedral structure, and thus brings unique benefits as a nanoparticle. The uses of these three plant viruses as either nanostructures or expression vectors for high value pharmaceutical proteins such as vaccines and antibodies are discussed extensively in the following review. In addition, the potential uses of geminiviruses in medical biotechnology are explored. The uses of these expression vectors in plant biotechnology applications are also discussed. Finally, in this review, we project future prospects for plant viruses in the fields of medicine, human health, prophylaxis, and therapy of human diseases.

## 1. Introduction

Over the past few years, plant viruses have increased in visibility for a wide range of applications in biotechnology. Plant viruses are highly suitable for production of vaccines as they are recognized by the innate immune system through the pathogen associated molecular pattern (PAMP) receptors [1] while being non-pathogenic to mammals. Plant viruses can elicit both cell-mediated immunity [2,3] and a humoral immune response when delivered through mucosal [4] or parenteral [5] routes.

**Figure 1:**
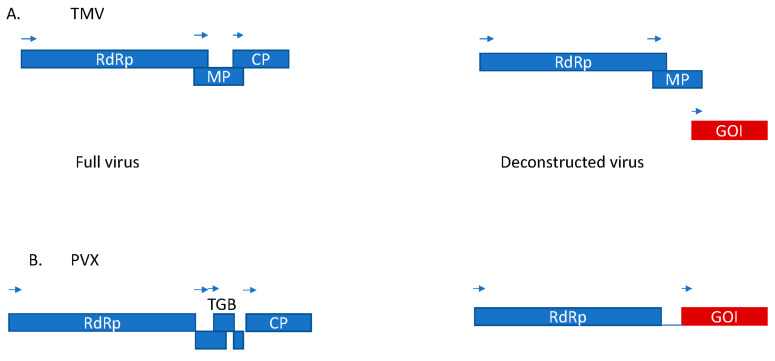
Schematic diagrams of full-length genome vs. deconstructed vectors of TMV (**A**) and PVX (**B**). RdRp, RNA-dependent RNA polymerase; MP, movement protein; CP, coat protein; GOI, gene of interest; TGB, triple gene block.

Plant virus genomes have been engineered to express heterologous open reading frames. For example, deconstructed virus vectors (Figure 1) were generated first using TMV [6,7] and PVX [8], of which the former was produced commercially by Icon Genetics as the magniCON vector [6]. Using this TMV magnifection technology, full immunoglobulin IgG was produced in under 2 weeks at high yields (4.8 g/kg fresh weight tissue) [9].

**Figure 2:**
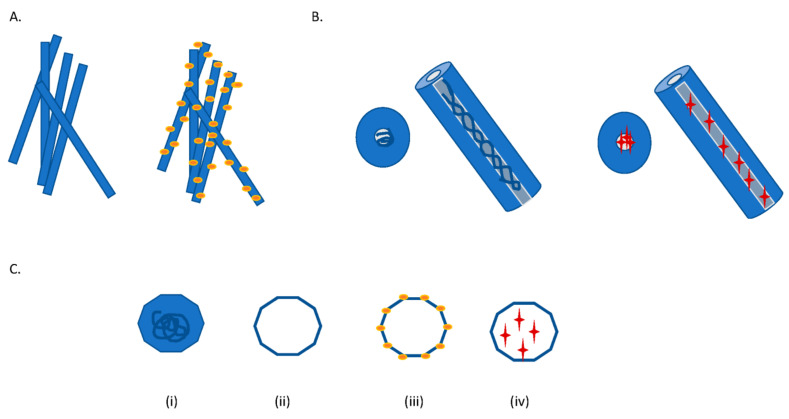
Schematic diagrams of TMV and CPMV wild-type virus vs. virus nanoparticles: (**A**) WT TMV (left hand side) and TMV nanoparticle (right hand side) displaying drug moieties conjugated to the surface of virus particle; (**B**) WT TMV (left hand side) containing viral RNA and TMV nanoparticle (right hand side), in this case RNA genome is replaced with drug moieties on the interior of the virus particle; (**C**) (**i**) CPMV intact virion, (**ii**) empty virus-like particle (eVLP), (**iii**) drug moieties conjugated to surface of eVLP, (**iv**) with drug moieties captured within eVLP.

Plant viruses have also been developed as VLPs and VNPs in order to present them as epitope display systems for vaccine production and as scaffolds for the conjugation of drugs or molecules used in diagnostics (Figure 2). VNPs and VLPs based on plant viruses are favorable because they are non-pathogenic to humans, and hence preclude any unwanted side effects/contamination. VNPs are nanoparticle formulations based on viruses that can be employed as building blocks for novel nanomaterials exhibiting a variety of molecular characteristics [10]. VNPs are self-assembling highly symmetrical systems that are dynamic, polyvalent, and monodisperse. They are advantageous due to reasons such as their robustness and ability to be generated in short time periods while serving as programmable molecular scaffolds. Additionally, VNPs are superior to synthetic nanomaterials by virtue of being biocompatible and biodegradable. Several self-assembly mechanisms have been adopted to encapsulate ligands such as small chemical modifiers, peptides, proteins, or even additional nanoparticles into the VNPs for which a wide range of conjugation chemistries have been employed [11,12]. These include strategies such as encapsulation, mineralization, chemical bioconjugation, and genetic engineering. 

VLPs are a subset of the VNPs but bereft of any nucleic acid genome, thus, making them noninfectious. VLPs are powerful vaccine candidates as they simulate the conformations of native viruses, utilizing their intrinsic immunogenicity while not compromising their safety. They accomplish this by having no viral genome, and therefore being unable to replicate [13]. Hence, VLPs have become popular as subunit vaccines while several plant viruses have been used to generate VNPs. VLPs evoke effectual immune responses as they are readily internalized by the antigen presenting cells (APCs) and are ideal platforms for antigen processing and epitope presentation to the immune system. Additionally, VLPs are increasingly used in cancer immunotherapy wherein their inherent ability to stimulate immune reactions can be employed to prime the tumor microenvironment towards launching antitumor immunity. VLPs occur as repetitive, multivalent molecular scaffolds by virtue of being composed of their capsid proteins in multiple copies that facilitate multivalent presentation of antigens. Therefore, VLP vaccines afford superior immunogenicity as compared with antigens in their soluble states. Additionally, plant viral VLPs and VNPs possess inherent adjuvant properties dispensing with the use of additional adjuvants to evoke strong immune responses. 

Knowledge and insight into the molecular structure of TMV [14] and PVX [15], for example, have enabled their use for several applications as biocatalysts [16], fluorescent markers [17], nanoparticles for in vivo imaging [18], nanoparticles for biologics purification [19], vaccines [20,21,22], and assembly units for memory devices [23]. Plant viral VNPs serve toward a variety of applications such as immunotherapy [24], chemotherapy [25], vaccines [26], gene delivery [27], and plant virus-assisted sensors [28].

The following review describes many of the uses of plant viruses in biotechnology, with examples based on TMV, PVX, CPMV, and geminiviruses. In the last section, we conclude with a future projection of the significance of plant viruses in the fields of medicine and engineering. 

## 2. Molecular Characteristics of TMV Advantageous for Biotechnological Use 

TMV was initially characterized in the 19th century and has since become a paradigm for our current perspective on the morphogenesis of self-assembling viral particle structures [29]. TMV is the most well-studied plant virus, and it is also the most important plant virus both scientifically and economically [30,31]. In recent times, this knowledge has been translated toward the generation of novel compounds and structures that could be used in nanotechnology and medicine. TMV can be easily produced and purified in bulk amounts, and therefore has become of tremendous importance in molecular biology and virology [32]. TMV has been used to detect translational enhancers for the augmented expression of heterologous genes [33,34], and for the design of effective vectors for virus-induced gene silencing and transient expression in plant systems [35], as well as for creating virus-resistant plant lines [36,37]. 

TMV is also simple and well-characterized with respect to particle structure and genome organization. Thus, it is well suited as a highly amenable experimental system for different applications. The rod-shaped virus particle measures 300 nm in length and 18 nm in diameter, and contains a 6.7 kb viral RNA genome that is encapsidated by 2130 identical copies of the capsid protein assembled in a helical arrangement. The crystal structure of the 158 amino acid capsid protein has been determined [38]. The genomic RNA contains a stretch of 432 nucleotide bases that forms the origin-of-assembly sequence (OAS) sufficient for viral assembly [39]. At neutral pH and without its RNA, the coat protein (CP) assembles itself into an 18 nm double disk, a 20S aggregate or nano-ring containing two layers of 17 CP molecules which can serve as a nanoscale scaffold. The amino acid sequence of the CP has many accessible regions for chemical modifications both at the inner and outer surfaces [30]. TMV can also assemble into spherical nanoparticles of 100–800 nm, in the absence of its RNA genome, by thermal processing [40]. Moreover, the TMV RNA genome can self-assemble with its purified CP in vitro to generate infectious virus particles [41], in addition to its ability to self-assemble in vivo. Therefore, TMV has become a model system for RNA-protein recognition. 

Different strategies can be used to modify TMV, such as the modification of the interior or exterior surface of the capsid through genetic engineering, chemical conjugation, or a combination of both processes. The interaction and transport of heterologous cargo within the virus inner cavity or generation of multivalent structures by particle integration have thus been adopted. The conformation of the TMV CP facilitates the insertion of foreign peptides at both its N- and C-termini. In addition to this, the loop formed from CP amino acids 59–66 can be used towards surface display of foreign peptides on intact virions or on CP assemblies [42]. 

## 3. The Use of Genetically Engineered TMV in Biochemistry, Nanotechnology, and Plant Biotechnology

Table 1 illustrates some examples of plant viral expression vectors derived from TMV [43,44,45,46,47,48], CPMV [49,50,51], PVX [52], and bean yellow dwarf virus (BeYDV) [53] for generation of foreign proteins. 

The location of C-terminus of the TMV CP on the exterior surface of assembled TMV virions makes it the most used site for insertion of foreign peptides. Table 2 presents some examples of the plant viruses (TMV [55,56,57], PVX [58], and CPMV [59,60]) used as drug delivery systems and the respective regions within their coat proteins that are amenable to genetic modifications. 

TMV particles have been exploited for active enzyme display, with wide-ranging uses in biodetection, sensor development, medicine, and enzymatic conversion. Enzymes such as penicillinase [62,63], horseradish peroxidase [64], and glucose oxidase [65] have been expressed on the TMV surface, as TMV exhibits a strong stabilizing effect on these enzymes. TMV adapter rods have been incorporated on sensor surfaces, which have facilitated bioaffinity-derived presentation of streptavidin conjugates of the above enzymes at surface densities that are not attainable on supports free of TMV. Enhanced reusability and augmented target detection ranges of these high-performance TMV-based biosensors have been reported and present great promise for multiple applications. 

TMV membranes have been engineered that could be recruited as tissue engineering frameworks by sequentially altered layering of two TMV variants with different charges. Recently, these TMV-based carrier templates have been used to prepare surfaces that promote cellular attachment and differentiation [66,67,68]. 

Some cells have been cultivated on TMV-covered culture supports and peptide ligands have been presented in a spatially defined manner over nanometric scales. Arginine–glycine–aspartic acid peptide associated TMV layers have been used for osteogenesis of stem cells from bone marrow [67,68]. TMV has been employed as a carrier for peptide motifs and is capable of cell-binding that simulates extracellular matrix proteins. TMV-derived nanorod fibers synthesized from complexation with electrospun composite polymers have been used to generate mats for better handling [69]. 

Transgenic plants expressing TMV CP were generated by Powell Abel et al. (1986) [37]. These plants showed resistance to TMV challenge and, as a result, initiated the theory of “capsid protein-induced resistance” [70]. TMV has also been used to engineer virus-induced gene silencing (VIGS) systems for *Colletotrichum acutatum*, a phytopathogenic fungus which proved to efficiently assemble virus particles inside hyphal cells [71]. 

## 4. The Use of TMV in Medicine, Cancer, Imaging, and Theranostics

TMV disks have a flat and round morphology that yields a high aspect ratio. TMV particles, by virtue of their flexuous rod-like structures, marginate toward blood vessel walls, enhancing the likelihood of invading diseased areas of the body, while accumulating inside tumor tissues [61,72]. In contrast to their spherical equivalents, the helical virus derived VLPs and VNPs transit more efficiently through tissues and membranes [73]. As compared with VLPs, VNPs are more effective because their RNA genome cargo functions as a ruler to define the length of the nucleoprotein–virus complex. In addition, the surface characteristics of these viruses can be altered by means of genetic or chemical approaches without compromising virus structural integrity. Consequentially, the positions of functional units such as drugs, contrast agents, or targeting ligands can be spatially controlled which enables the engineering of multifunctional systems that harbor different combinations of these moieties [74]. 

Molecular imaging is an emerging biomedical field which facilitates the visualization, identification, and evaluation of biological mechanisms in vivo. Some of these imaging technologies include magnetic resonance imaging (MRI), computed tomography (CT), positron emission tomography (PET), and optical imaging, which enable the monitoring of molecular and cellular processes in normal and diseased conditions in living subjects. Ideally, a given molecular imaging technique should readily afford optimal signal-to-noise ratios within the target site while minimizing toxicity [13]. 

VLPs are more beneficial for molecular imaging technologies than synthetic nanoparticles, due to their short half-life in circulation and their low retention times, which thus reduce probable side effects [10]. Furthermore, VLPs can be developed to carry a wide array of contrast agents and fluorescent labels, as they can be modified with antibodies, peptides, and aptamers to enable enhanced targeting to specific tissues and cells. 

TMV has been successfully used for imaging, targeting atherosclerosis, and thrombosis [75]. Cargo mRNA encoding the green fluorescent protein (GFP) was encapsulated within TMV, which when administered into mice, elicited an immune response against GFP. This provided a proof-of-concept that this technology can be utilized for vaccine development [76]. TMV has also been engineered to display the iLOV protein, which acts as a fluorescent probe [77]. TMV has also been used in theranostics (drugs and/or techniques combined to both diagnose and treat medical conditions), for enabling photoacoustic imaging and MRI capabilities to photothermal therapy (PTT) treatment. 

Table 3a presents some examples of the use of the engineered TMV for treating diseases, while Table 3b shows a list of studies wherein TMV has been used for cancer treatment. Table 3c presents examples of studies using TMV in theranostic applications.

## 5. Molecular Characteristics of PVX Advantageous for Biotechnological Use

PVX is a single-stranded, positive-sense RNA virus with a flexuous rod-like morphology. The PVX genome is 6430 bases in length [101] and contains a 5′ cap structure and 3′ poly-A tail. There are five open reading frames (ORFs) encoding the ORF1 replicase protein for viral replication, the ORF 2, 3, and 4 triple gene block (TGB) proteins which mediate virus movement and the ORF5 capsid protein for encapsidation and cell-to-cell movement. Protein overexpression systems based on plant viruses are more economical and easier to implement as compared with stable transformation which is more laborious and could take protracted lengths of time [102], whereas infecting plants with genetically engineered viruses directly or through Agrobacterium-mediated infiltration enables easy, rapid, highly efficient transient expression of heterologous proteins. Particularly, the sequence between the TGB and the CP can be modified to clone and express foreign genes [8,103,104]. 

## 6. PVX as an Expression Vector and Repurposing PVX for Use in Medicine, Cancer, and Theranostics

PVX has been widely explored as an expression vector for several biopharmaceutical applications such as for antigenic epitopes displayed on the virus outer surface, as well as for expressing full-length and fusion proteins [105]. Virus-derived biocatalysts have been generated using filamentous PVX that was integrated with the enzyme lipase [16]. The major advantage of this scaffold is the ability of the PVX-lipase complex to self-replicate, unlike the equivalent synthetic systems. Such enzymes can be positioned in or on the virus capsid, thus, spatially combining several different enzymes into specific groups that can simulate metabolic cascades. 

Of note is the engineering of PVX to serve various biomedical purposes. Uhde-Holzem et al. (2016) [106] reported genetically altered PVX which displayed *Staphylococcus aureus* protein A fragments on its surface, and proved to be easily functionalized with IgG to be used in biosensing plant viruses [107]. PVX has also been widely used in biotechnology, disease diagnostics, development of vaccines/antibodies against infectious diseases, as well as cancer research and treatment. The CP of PVX is not capable of forming VLPs on its own [108,109]. PVX nanoparticles have been shown to inhibit tumor growth in both cell lines and animal models [110]. They are increasingly being used for immunotherapy of tumor microenvironments.

PVX-based VLPs and VNPs are ideal tools in molecular imaging and unlike synthetic nanoparticles, they have limited half-lives in circulation as well as diminished retention times, thereby, decreasing the chances of unwanted side effects. Additional studies have reported that PVX has been conjugated to fluorescent reporters that could be applied towards theranostics, nanomedicine, and in vivo imaging [111]. The small fluorescent iLOV protein was expressed on PVX through genetic engineering, and the resultant engineered PVX served as a fluorescent probe which could be of potential use in vivo imaging. Shukla et al. (2018) [112] reportedly produced PVX VNPs that displayed mCherry or GFP on their N-termini in *N. benthamiana* plants. Significantly, fluorescent PVX could successfully be used for in vivo particle tracking in an HT-29 murine model, for in vitro imaging of HT-29 cells, and for tracing viral infection within plants. 

In plant systems, PVX has been used in the identification of pathogenicity determinants of various viruses, fungi, and bacteria (Table 4a). Table 4b presents examples of studies using PVX for diagnosis, prophylaxis, and therapy of infectious diseases, while Table 4c shows instances where PVX has been successfully used in the treatment of cancer. 

## 7. Molecular Characteristics of CPMV Advantageous for Biotechnological Applications

CPMV is the type member of the genus *Comovirus*, composed of two separately encapsidated positive-strand RNAs. RNA-1 is capable of independent replication in plant cells; however, RNA-2 (encoding the viral movement and structural proteins) depends on RNA-1 for its replication. CPMV virions are icosahedral in shape and are comprised of 60 copies each of a large (L) and a small (S) coat protein [129]. 

## 8. Applications of Comoviruses CPMV and Cowpea Chlorotic Mottle Virus (CCMV) in Medical Biotechnology and Cancer 

CPMV has been developed as an autonomously replicating virus vector for the expression of either peptides or polypeptides in plants (Table 5). Examples of CPMV used as an epitope presentation system include epitopes from the outer membrane (OM) protein F of *Pseudomonas aeruginosa* which were shown to protect mice against bacterial challenge, and an epitope expressing the 30 amino acid D2 domain of the fibronectin-binding protein (FnBP) from *Staphylococcus aureus*, which has been shown to be able to protect rats against endocarditis [130]. 

In addition to the use of CPMV to present peptides, replicating and non-replicating expression vectors based on CPMV have been developed [131]. The non-replicating expression system is based on a disabled version of RNA-2 of CPMV. A gene of interest is positioned between the 5′ leader sequence and 3′ untranslated region (UTR) of RNA-2, and the vector is introduced to the plant via Agrobacterium-mediated transient transformation [50]. By deleting an in-frame initiation codon located upstream of the main translation initiation site of RNA-2, a massive increase in foreign protein accumulation has been observed. This CPMV non-replicating system generated high quality purified anti-HIV-1 antibody in plants [132]. The vector has also been used to express influenza vaccine proteins. 

Meshcheriakova et al. (2017) compared the differences between empty virus-like particles (eVLPs) of CPMV and intact virus containing its RNA genome, for their potential use as nanoparticles [133]. eVLPs are noninfectious and could be loaded with heterologous material, which has increased the number of possible applications for CPMV-based particles. In addition to this, they have distinct yet overlapping immunostimulatory effects resulting from virus RNA in wild-type particles, and therefore can be used for different immunotherapeutic strategies [134]. 

As described for TMV, CPMV has been explored for its potential to block cancer [135]. Steinmetz et al. (2011) found that CPMV nanoparticles could bind to vimentin, a protein found on the surface of most cells [136]. Vimentin is upregulated during tumor progression, making it an attractive target for cancer therapy. The fact that surface vimentin expression correlated with CPMV uptake in this study demonstrated the ability of CPMV to detect invasive cancer cells. Soon after this discovery, Lizotte et al. (2016) found that inhaled CPMV nanoparticles could be rapidly taken up by lung cancer cells in a mouse model and activated neutrophils in the tumor microenvironment to initiate an antitumor immune response [137]. CPMV nanoparticles also demonstrated antitumor immunity in ovarian, colon, and breast tumor models in mice. 

Patel et al. (2018) used CPMV nanoparticles in conjunction with radiotherapy to delay ovarian tumor growth in a mouse model [138]. The treatment was able to result in an increase in tumor infiltrating lymphocytes (TILs), suggesting that this combined treatment could act as a future in situ tumor vaccine. Further studies by Wang and Steinmetz (2019) found that a protein known as CD47, which is widely expressed on tumor cells, prevents the action of T cells and phagocytic cells. The authors used a combination therapy of CD47-blocking antibodies and CPMV nanoparticles to act synergistically and elicit an antitumor immune response [139]. The same research group also used low doses of cyclophosphamide (CPA) and CPMV nanoparticles as a combination therapy to successfully reduce mouse tumors in vivo [140]. 

Recently, Albakri et al. (2019) explored how CPMV particles could activate human monocytes, dendritic cells (DCs), and macrophages [141]. Monocytes, upon incubation with CPMV in vitro, released the chemokines CXCL10, MIP-1α, and MIP-1β into cell culture supernatants. Dendritic cells and monocyte-derived macrophages also were activated after incubation with CPMV. The authors found that activation was part of SYK signaling. Shukla et al. (2020) were able to demonstrate that CPMV outperformed many other types of virus-like particles, and therefore was a particularly strong immune stimulant [142]. 

Plant VLPs based on CCMV have been employed to deliver mRNA. For example, CCMV was used to successfully deliver enhanced yellow fluorescent protein (EYFP) mRNA to mammalian BHK-21 cells, using transfection with lipofectamine. In this case, the mRNA was successfully delivered and released from the VLPs into the cytoplasm of the BHK-21 cells, facilitating EYFP expression [27]. Furthermore, CCMV can be used to deliver mRNA vaccines, and a proof of concept has been demonstrated with a variety of reporter genes [143]. 

There are other examples of how icosahedral VLPs can be utilized in medicine. For example, CCMV can be disassembled and reassembled to encapsulate CpG ODNs (oligodeoxynucleotides). CpG ODNs are ligands of the toll-like receptor 9 (TLR9). Upon activation, TLR9 has the capability to induce macrophages. The CpG loaded CCMV VLPs showed significantly enhanced uptake by tumor associated macrophages and inhibited the growth of solid CT26 colon cancer and B16F10 melanoma tumors in Balb/c mice via the macrophage activation [144].

As another example, encapsulated drug-activating enzymes within plant VLPs such as CCMV can be utilized for therapeutic purposes [145]. Cytochrome P450 family enzymes can convert chemotherapeutic prodrugs into an active format. Using plant VLPs to encapsulate these enzymes can reduce side effects while increasing retention and targeting to the tumor site [25,146]. CCMV has been used, for example, to encapsulate bacterial cytochrome, CYPBM3, to activate the prodrugs into activated forms of tamoxifen and resveratrol.

## 9. Molecular Features of Geminiviruses Advantageous for Biotechnological Use

Plant viruses with ssDNA genomes offer an exceptional alternative format for expression vector design. These plant viruses tend to have small genomes that can readily incorporate open reading frames of unrestricted sizes. They replicate using a rolling circle mechanism and can express genes of interest at extremely high levels; they also infect a broad range of different plant varieties. Geminivirus constructs, for example, require only the virus origin of replication, the gene of interest, and the replication-associated protein (Rep) gene provided in cis or trans format for potential expression in a wide range of plant families [147,148]. Geminiviruses are considered unique for their twinned capsid morphology. Although they are transmitted in the wild by insects, they are readily amenable to genetic engineering and can be introduced easily into plants in a laboratory setting.

## 10. The Use of Geminiviruses in Biotechnology and Medicine

Bean yellow dwarf virus (BeYDV) is a geminivirus frequently used for expression of pharmaceutical proteins. BeYDV has recently been used to produce norovirus, HIV, HPV, and hepatitis B virus subunit vaccines, monoclonal antibodies to West Nile virus and Ebola virus, as well as earthworm-derived Lumbrokinase (PI239), used to dissolve fibrin and blood clots [149,150]. Besides using higher plants such as tobacco as hosts, geminiviruses have also been used to express proteins in algae [151]. In this case, a microalgae-based system known as Algevir was utilized to produce Ebola virus vaccine protein as well as the highly immunogenic B subunit of the heat-labile *Escherichia coli* enterotoxin. The authors generated a yield of 1.25 mg/g fresh biomass (6 mg/L of culture), within 3 days after transformation. 

More examples of the use of geminiviruses for pharmaceutical production include the expression of plant-made recombinant immune complex (RIC) vaccines [152,153]. In one instance, a bio-better vaccine toward Zika virus (ZIKV) was established. The antigen fusion site ZE3 on the RIC platform was altered to accommodate an N-terminal fusion to the IgG heavy chain (N-RIC) with an improvement of 40% in RIC expression. This construct produced a strong antibody titer that correlated with neutralization of the Zika virus. Moreover, when these RICs were co-delivered with plant-produced hepatitis B core (HBc) virus-like particles (VLP) displaying ZE3, there was a five-fold greater antibody titer (>1,000,000) that more strongly neutralized ZIKV than using either RICs or VLPs alone, in the absence of adjuvant and after only two doses [154].

In another recent study, a variety of plant-made human IgG1 fusion vaccine candidates were examined using Zika virus (ZIKV) envelope domain III (ZE3) as a model antigen. These fusion constructs were altered to make RICs and generated using geminivirus vectors in plants which had their glycosylation pathways altered to make the plant more humanized in its glycan profile. The results of this study were the generation of a vaccine candidate at 1.5 mg IgG fusion per g leaf fresh weight that generated high titers of antibodies specific for Zika virus [155].

Future directions for use of geminivirus expression vectors follow the blossoming new field of genome editing, with this expression vector carrying CRISPR/Cas9 machinery to enable precise gene editing through homologous recombination [156]. 

**Table 5:** Medical applications of comovirus and geminivirus vectors.

Virus	Application	References
Comovirus CPMV	Delays tumor growth using combination therapy	[137,138]
	CPMV and cyclosposphamide	[140]
	Activation of monocytes, dendritic cells, macrophages	[141]
Comovirus CCMV	mRNA vaccine delivery	[143]
	Encapsulate CpG oligonucleotides, activated macrophages and inhibit growth	[144]
	Encapsulate drug-activating enymes to reduce side effects, increase targeting to tumor site	[145,146]
Geminivirus BeYDV	Vaccines and monoclonal antibodies	[149]
	Monoclonal antibodies to West Nile Virus, Ebola Virus	[149]
	RIC vaccines to ZIKV	[154,155]

## 11. Viral Expression Vectors and the CRISPR/Cas9 Technology

The agricultural industry has been greatly burdened by infections due to plant viruses and several genetic engineering techniques have been applied to confront plant viral infections. Since the turn of the century, RNA interference has been used effectively for this purpose. In a reported pioneering investigation by Zhang et al. (2018), FnCas9 from Francisella novicida and its guide RNA were used to target the RNA genome of TMV and cucumber mosaic virus (CMV) to engineer virus resistance [157]. Three sites of the TMV genome were targeted which inhibited virus accumulation by 40–80%. Additionally, it was found that the FnCas9 bound the RNA genome, but did not cleave it, thus, limiting the chances of the emergence of viral escape mutants and facilitating durable resistance towards virus control in the long term. 

Ariga et al. (2020) reported the use of a PVX vector expressing the cas9 gene and single-guide RNA for highly effective targeted mutagenesis in the model system, *N. benthamiana* [158]. The virus vector was introduced through Agrobacterium transformation that enabled transgene-free gene editing. On the one hand, this coupled with high level expression by amplification of the viral RNA, wherein the PVX can accommodate the large size of the Cas9 gene, is of great applicability in precise editing of the plant genome. On the other hand, other viruses such as the TMV, beet necrotic yellow vein virus, and the tobacco rattle virus cannot accommodate the Cas9 gene due to their size limitations and are known to work only with the Cas9 applied in trans. Deconstructed geminiviruses have been used to express Cas9 successfully, however, such deconstructed forms are not infectious.

One of the most significant antiviral mechanisms of plants is RNA silencing. This is executed through the essential function of the small RNA guided Argonaute proteins which act as agents of viral restriction. One of these proteins is AGO2 which has been proven to be involved in antiviral responses in the host Arabidopsis thaliana. In a study by Ludman et al. (2017), the role of AGO2 in conferring antiviral immunity was explored using *Nicotiana benthamiana* as the host plant [159]. In this investigation, the CRISPR/Cas9 technology was used to inactivate the AGO2 gene which plays an important role in the immune responses of the plant against PVX and other plant viruses.

## 12. Conclusions

During the 1980s, the brome mosaic virus (BMV) and the cauliflower mosaic virus (CaMV) were genetically engineered as the first RNA and DNA plant virus vectors, respectively, to express bacterial genes [160,161]. Since then, several vectors based on plant viruses have been designed as efficient tools for the expression of recombinant proteins and to advance genomic research. Thus far, many plant viruses have been recruited as delivery vectors for several purposes. These include viruses infecting dicotyledonous plants such as potexviruses [8,162,163,164], tobamoviruses [165,166], furovirus [167], potyvirus [168,169,170,171,172], geminiviruses [171], comoviruses [172,173], Necrovirus [174], and Caulimovirus [161]. Further, viruses such as Foxtail mosaic virus [175], barley stripe mosaic virus [176,177,178], wheat streak mosaic virus [179] and soil-borne wheat mosaic virus [167] capable of infecting monocotyledonous plants have been repurposed as expression vectors. Plant viral expression vectors are increasingly being used in basic and applied research requiring the expression of pharmaceutical peptides, antibodies, and other functional complex heterologous proteins. Furthermore, these vectors have been used in functional genomics applications such as virus-based miRNA expression, VIGS, identification of virulence effectors, and virus-mediated genome editing. This review discusses the use of the most popular plant viruses namely the TMV, PVX, CPMV, and geminiviruses for biotechnological purposes, medicine, and human health. While this is not by any means exhaustive considering the wealth of recent and older literature in this area, it addresses some of the major achievements in the use of these viruses as expression vectors.

In the current review, we highlight the use of plant virus based VLPs and VNPs as diagnostic and therapeutic agents for biotechnological and biomedical applications such as VIGs, identification of virulence effectors of plant pathogens, vaccines against cancer and infectious diseases, theranostics and nanocarriers for imaging modalities. VNPs and VLPs play a major role in the future of nanotechnology and nanomedicines. Viruses and VNPs are natural carriers of nucleic acid molecules which protect and transport their cargo, and this is the major property used for drug delivery. Through a combination of chemistries and by attachment of a wide range of functional groups, drug cargo can be encapsulated, infused, conjugated, or absorbed to the exterior and interior surfaces of their coat protein interfaces [180]. This affords molecular flexibility towards protection of cargo with proteinaceous matrices, reversible binding of active molecules, and specific targeting to the sites of action. VNPs are advantageous as natural delivery carriers because of their structural uniformity, water solubility, biocompatibility, ease of functionalization, and high uptake efficacy [181]. Nanosized cages afford ideal approaches for imaging and drug delivery while conferring high stability, cell-targeting, cell penetrability, and appropriate pharmacokinetics. In addition, VNPs do not show tissue tropisms, and therefore can be employed for targeting and binding cell surface receptors, crossing membranes and penetrating the nucleus [182].

The structures of several viruses are known at atomic resolution enabling modifications with spatial selectivity in a precise manner. By genetic engineering, the VLPs can be formulated to obtain new structures having predictable interactions with biological systems]. VLPs can be engineered to display on their surface functional groups such as ligands for targeting, epitopes, imaging dyes, and drug payloads. The VLPs by virtue of their size and shape facilitate vascular transport, active cellular uptake, and molecular interactions. VLPs can tolerate harsh environments while being biocompatible. In addition, high doses of VLPs are mostly well tolerated and the VLPs are completely and rapidly cleared by proteolytic degradation to enable diminished side effects. Moreover, the characteristic ability of the VLPs to self-assemble coupled with novel molecular design using chemical biology technologies enable the production of functionalized hybrid VLP nanomaterials. The field of VNP- and VLP-based technologies for drug delivery applications continues to evolve with several candidates in clinical trials that should lead to advanced therapeutics, in the near future. In the future, it is very much likely that more plant viruses would be genetically engineered and repurposed for further use in biotechnology and medicine.

## Figures and Tables

**Table 1:** Examples of plant viruses used as expression vectors for foreign proteins.

Recombinant Protein or Vaccine or VLP	Viral Vector
Cholera toxin b subunit	TMV [43]
Human anti-non-Hodgkin’s lymphoma single-chain Fv (scFv) immunoglobulins	Hybrid TMV and odontoglossum ringspot virus (ORSV) [44]
Rice a-amylase	Hybrid TMV and tomato mosaic virus (ToMV) [45]
Assembled full-size monoclonal antibody	Combination of non-competing viral vectors TMV and PVX [46]
Human growth hormone	Hybrid crucifer-infecting TMV (cr-TMV) and turnip vein-clearing virus (TVCV) [47]
Plant-produced VLP developed for drug delivery	TMV [48]
Plant-produced chimaeric virus vaccine for influenza virus	TMV [21]
Assembled full-size monoclonal antibody	CPMV [49]
Plant-produced chimaeric virus vaccine for human rhinovirus 14 and human immunodeficiency virus	CPMV [50]
Plant-produced VLP developed for encapsulation of metals	CPMV [51]
Plant-produced chimaeric virus vaccine for hepatitis C virus	PVX [52]
Hepatitis B core Norwalk virus capsid protein (NVCP)	BeYDV [53]

(Adapted from Ibrahim et al., 2019 [54]).

**Table 2:** Examples of plant viruses used in drug delivery systems.

Virus	Symmetry	Family	Locations within the CP Amenable to Genetic Modification
TMV	Rod-like	Tombusviridae	Threonine 104/158, serine 123, N/C-terminal of coat protein [55,56,57]
PVX	Rod-like	Potexviridae	N-terminal of coat protein [58]
CPMV	Icosahedral	Comoviridae	βB-βC loop of the small subunit/βE-βF loop of the large subunit [59,60]

(Adapted from Sokullu et al., 2019 [61]).

**Table 3:** Applications of TMV in biotechnology and medicine: (**a**) Applications of TMV in medicine; (**b**) applications of TMV in cancer treatment; (**c**) applications of TMV in theranostics.

**(a) Applications of TMV in medicine**		
**Engineered Modifications**	**Effects **	**Reference**
The extreme C-terminus of the TMV CP fused to the 11 amino acid epitope of the foot and mouth disease virus (FMDV) VP1 protein	This nanoparticle protected animals against FMDV challenge	[78]
Peptides from the coronavirus murine hepatitis virus spike protein displayed on the surface of TMV particles	Increased antibody titers and protected mice against murine hepatitis virus challenge	[79]
An epitope from Pseudomonas aeruginosa outer membrane protein F fused to the C-terminus of the TMV CP	Demonstrated immunity to Pseudomonas aeruginosa	[80]
The influenza virus M2e epitope displayed by fusion near the C-terminus of the TMV CP	Afforded protective anti-influenza immune response in mice	[21]
TMV conjugated to the thrombolytic tissue plasminogen activator tPA	Functioned efficiently equivalent to free tPA and enhanced safety profile as shown by diminished average bleeding times and therefore applicable for cardiovascular therapy	[81]
**(b) Applications of TMV in cancer treatment**		
**Engineered Modifications**	**Effects**	**Reference**
TMV employed to display a weakly immunogenic tumor-associated carbohydrate antigen, the Tn antigen (GalNAc-α-O-Ser/Thr)	Potent immune responses were observed when the Tn antigen was conjugated to Tyr 139 of TMV	[82]
TMV CP used as nanocarrier for a highly hydrophobic, insoluble peptide that binds to the neuropilin (NRP1) receptor transmembrane domain in cancer cells	Shown to be anti-angiogenic by reducing cancer cell growth and migration	[30]
Doxorubicin (DOX) loaded onto TMV disks	Increased rates of survival of mice bearing intracranial glioblastoma	[83]
DOX loaded onto TMV VNPs coated with albumin	Antitumor effects	[84]
Cisplatin and phenanthriplatin loaded into the cavity of TMV by formation of stable covalent adduct or by charge-based reaction	Enhanced absorption by cancer cells and improved cytotoxicity	[85,86]
TMV VNPs loaded with cisplatin modified using lactose and mannose moieties on their external surface	This construction assisted the VNP’s recognition by the asialoglycoprotein receptor that is present on cell membranes and demonstrated augmented cytotoxicity in cancer cell lines	[87]
Modification of the TMV coat protein with a molecular fluorous ponytail incorporated at specific sites which resulted in self-assembly of the virus into spherical VNPs	These spherical VNP’s conferred greater stability of for the cisplatin-VNP complexes formed via metal-ligated coordination	[88]
Mitoxanthrone (MTO) loaded onto TMV VNPs by a charge-driven mechanism	Increased antitumor effects in mice	[89]
Antimitotic drug, valine-citrulline monomethyl auristatin E loaded onto external surface of TMV VNPs	Effective targeting and cytotoxicity in non-Hodgkin’s lymphoma cell line, Karpas 299; internal entry of TMV VNPs into endolysosomal components accompanied by protease-encoded release of the drug	[90]
Transacting activation transduction (TAT) peptide fused to the external surface of TMV	The engineered TAT-tagged TMV was internalized; this delivered RNA silencing in nude mice hepatocellular carcinoma tumors upon intravenous and intratumoral delivery	[91]
Zn-EpPor (5-(4-ethynylphenyl)-10,15,20-tris(4-methylpyridin-4-ium-1-yl)porphyrin-zinc(II) triiodide), a photosensitizer drug loaded onto the interior of the TMV particles	Demonstrated high stability and shelf-life; drug was released into endolysosomes and showed augmented cell-killing efficiency	[61]
Zn-Por+3 loaded TMV conjugated to F3 peptide	Targeted the nucleolin shuttle protein overexpressed on Hela cells; drug accumulated on cell membranes along with increased cell-killing efficiency likely due to disruption of the cell membrane through light activation followed by drug release and cellular uptake	[92]
**(c) Applications of TMV in theranostics**		
**Engineered Modifications**	**Effects**	**Reference**
A near infrared fluorescent (NIR) dye as well as a peptide targeting S100A9 (a myeloid-related protein 14 present in atherosclerotic lesions and a molecular marker for acute myocardial infarctions) were conjugated to TMV	These targeted TMV particles were able to identify atherosclerotic lesions in apolipoprotein E-deficient (ApoE-/-) mice upon intravenous injection, showing that TMV can be used as a platform to detect at-risk lesions	[75]
A TMV-MOF (metal-organic framework) hybrid nanoparticle engineered	Increased retention of the TMV VNPs observed in mice	[93]
A Cy5-encapsulated TMV coated with zeolitic imidazolate framework-8 (Cy5-TMV@ZIF)	Improved the fluorescence retention time by 2.5 times more than that of the Cy5-TMV alone; this TMV@ZIF was recalcitrant to harsh conditions and proved to be highly stable and non-toxic	[93]
Gd-dodecane tetraacetic acid (Gd-DOTA) loaded onto TMV particles altered to target the vascular cell adhesion molecule, VCAM-1	Facilitated the sensitive identification and depiction of atherosclerotic plaques in ApoE-/- mice, using low doses of the contrast agent wherein the augmented relaxivity and slower tumbling of the Gd-DOTA coupled with the TMV carrier improved the signal-to-noise ratio; also, this coupling afforded greater sensitivity of imaging, allowing 40× decrease in Gd dose in comparison with the standard clinical doses	[94]
Packing of a dysprosium (Dy3+) complex within the interior cavity of TMV	Enhanced T2 relaxivity towards MRI; this enabled NIR fluorescent dye delivery, which facilitated dual optical-MR imaging. The exterior surface of TMV was labeled with an Asp-Gly-Glu-Ala peptide that enabled target specificity to integrin α2β1 molecules on prostate cancer cells	[13]
A metal-free paramagnetic nitroxide organic radical contrast agent (ORCA) loaded onto TMV particles to generate electron paramagnetic resonance and MRI probes towards the detection of superoxide	This augmented in vitro r1 and r2 relaxivities and these probes worked as both T1 as well as T2 contrast agents, facilitating their suitability for preclinical and clinical MRI scanning	[95]
TMV conjugated to a derivative of the aminoxyl radical TEMPO (tetramethylpiperidin-1-oxyl, coined Compound 6) by means of a copper catalyzed azide-alkyne cyclo-addition reaction	Subsequent interaction with cucurbit [8] uril (CB [8]) generated an aminoxyl-based ORCA (semitroxane) that was silent for MRI; the r1 (relaxivity) values for TMV-6 emulated that of Gd-DOTA	[96]
TMV nanorods loaded with Gd and coated with polydopamine (PDA)	The PDA enhanced the MRI properties and provided PDA contrast, while simultaneously facilitating photothermal therapy (PTT); strong in vitro NIR absorption was observed along with increased photothermal conversion efficiency, compared to that of gold nanocages [97] and nanorods [98]; also, these VNPs demonstrated potent efficiency with lowered cytotoxicity in treating 4T1 breast and PC-3 prostate cancer cells in vitro	[99,100]

**Table 4:** Applications of PVX in biotechnology and medicine: (**a**) Applications of PVX in identifying pathogenicity determinants and in VIGS; (**b**) applications of PVX in the diagnosis, prophylaxis and therapy of infectious diseases; (**c**) applications of PVX in cancer.

**(a) Applications of PVX in identifying pathogenicity determinants and in VIGS**		
**Engineered Modifications **	**Effects **	**Reference**
PVX used as an expression vector for the production of V2, C1, and C4 proteins of a novel monopartite begomovirus, the Ageratum leaf curl Sichuan virus in N. benthamiana	Deletion and mutational analysis of the C4 protein using this PVX-derived vector showed that C4 is the major pathogenicity determinant which impacted symptom expression and virus accumulation	[113]
Phytophthora sojae virulence effector Avh148 expressed in plants using a PVX-based vector and a virus-induced virulence effector (VIVE) assay to detect putative effectors encoded by various plant pathogens	This PVX-Avh148 vector infected plants with strong viral symptoms and led to elevated levels of Avh148 effector and viral RNA accumulation; Avh148 was found to be essential for full pathogenic virulence; this VIVE assay could detect putative effectors encoded by various plant pathogens including even unculturable pathogens using this PVX-based expression vector	[114]
Grapevine leafroll-associated virus 2 (GLRaV-2) encodes a p24 polypeptide (a suppressor of RNA-silencing) that was expressed in a PVX-based vector	p24 causes systemic necrosis in N. benthamiana wherein a cytoplasmic Zn2+-binding protein, NbRAR1 is involved and the symptoms are characteristic of a hypersensitive response; the essential role of p24 in GLRaV-2 pathogenesis was elucidated using the PVX expression vector wherein both silencing suppression and p24 self-interaction are critical for the pathogenic activity of p24	[115]
Tomato torrado virus (ToTV) capsid protein subunits Vp23, Vp26, and Vp35 expressed transiently from a PVX-derived vector in Solanum lycopersicum	Of these, Vp26 protein was shown to be the necrosis and pathogenicity determinant responsible for severe systemic necrosis of the plants accompanied by increased ribonuclease and oxidative activities	[116]
PVX has been developed as a VIGS vector in potatoes wherein VIGS mediates silencing of endogenous plant genes, thus helping to investigate the functions of the silenced genes	This caused the silencing of the endogenous phytoene desaturase gene in potato plants which led to characteristic photobleaching symptoms in the leaves by interference of the carotenoid biosynthetic pathway	[117]
**(b) Applications of PVX in the diagnosis, prophylaxis, and therapy of infectious diseases**		
**Engineered Modifications**	**Effects**	**Reference**
The scFv-TM43-E10 and scFv-Fc-TM43-E10 antibody derivatives specific for the recognition of the Salmonella typhimurium Omp D protein expressed in a deconstructed PVX vector deficient for virus movement	These PVX vector-based antibodies exhibited similar antigen-binding specificities as that of their mammalian/microbial cell-generated counterparts and were able to successfully recognize the *S. typhimurium* Omp D antigen; therefore showed great promise as new diagnostic tools for the detection of *S. typhimurium* infection	[118]
The Severe Acute Respiratory Syndrome Coronavirus (SARS-CoV) N and M proteins expressed using PVX	The presence of antibodies specific to the SARS-CoV N protein could be detected in SARS-CoV patient sera using the plant-derived N protein	[119]
The M2e peptide of H1N1 Influenza virus was fused to bacterial flagellin to augment immunogenicity and then expressed in a PVX vector	The yield of the fusion protein was as high as 30% of the total soluble protein and mice inoculated with the PVX-derived protein exhibited protection against Influenza virus infection	[120]
The hyper variable region 1 (HVR-1) epitope of Hepatitis C Virus (HCV) expressed in a PVX Vector and administered parenterally	This elicited IgG immune response and the PVX-HVR1 epitope reacted positively with the serum of chronic HCV patients	[53]
A second capsid protein promoter of PVX used to express a chimaeric protein derived from fusion of the HCV core antigen with the hepatitis B virus (HBV) surface antigen (HBsAg)	This PVX-based polytopic HCVpc-HBsAg construct could be a potential plant-derived HCV vaccine	[121]
**(c).Applications of PVX in cancer**		
**Engineered Modifications**	**Effects**	**Reference**
PVX used as an expression vector for Mambalgin-1, a peptide that functions as a potent analgesic by obstructing acid-sensing ion channels (ASIC) in nerve cells wherein the ASIC is involved in the growth and proliferation of cancer cells	This resulted in the production of Mambalgin-1 which exhibited cytotoxicity towards nervous (SH-SY5Y) cancer cells, inhibited ASIC channels and potentiated anticancer effects	[122]
Monoclonal antibodies of Herceptin or Trastuzumab loaded onto PVX nanofilaments	This successfully induced apoptosis in breast cancer cell lines	[123]
PVX used as an expression vector for a mutant form of the HPV16 E7 oncoprotein, by fusing it with lichenase	This elicited protection against tumor progression in mice by inducing robust cytotoxic T-cell response	[124]
The filamentous PVX used to deliver DOX	These DOX-loaded PVX VNPs greatly diminished the growth of tumors in athymic mice harboring breast cancer xenografts	[125]
PVX-DOX combination	Prolonged mouse survival and stimulated chemokine/cytokine levels in mouse intradermal melanoma models	[126]
PVX used to display tumor necrosis factor-related apoptosis inducing ligand (TRAIL)	Multivalent display of TRAIL enabled increased recruitment and stimulation of death receptors expressed on cancer cell lines and successfully suppressed tumor growth in mice breast cancer models	[127]
PVX conjugated to an idiotypic (Id) tumor-associated antigen (TAA) recombinant through a biotin/streptavidin linker	This elicited a 7 times higher anti-Id IgG response as compared with Id alone in a mouse B-cell lymphoma model; IFN-α and IL-12 were induced; also TLR7 was found to be essential for viral RNA recognition	[128]

## Data Availability

Not applicable.
